# The Conundrum of Volume Status Assessment: Revisiting Current and Future Tools Available for Physicians at the Bedside

**DOI:** 10.7759/cureus.15253

**Published:** 2021-05-26

**Authors:** Mohammed G Elhassan, Peter W Chao, Argenis Curiel

**Affiliations:** 1 Internal Medicine, Saint Agnes Medical Center, Fresno, USA

**Keywords:** volume status assessment, hypovolemia, hypervolemia, physical examination, bedside ultrasound, euvolemia, point-of-care ultrasound

## Abstract

Assessment of patients’ volume status at the bedside is a very important clinical skill that physicians need in many clinical scenarios. Hypovolemia with hypotension and tissue under-perfusion are usually more alarming to physicians, but hypervolemia is also associated with poor outcomes, making euvolemia a crucial goal in clinical practice. Nevertheless, the assessment of volume status can be challenging, especially in the absence of a gold standard test that is reliable and easily accessible to assist with clinical decision-making. Physicians need to have a broad knowledge of the individual non-invasive clinical tools available for them at the bedside to evaluate volume status. In this review, we will discuss the strengths and limitations of the traditional tools, which include careful history taking, physical examination, and basic laboratory tests, and also include the relatively new tool of point-of-care ultrasound.

## Introduction and background

The case

An 83-year-old African American female presented to the emergency department (ED) with worsening fatigue, dyspnea, and mild cough. She had no prior history of cardiac or lung disease. Her vital signs were normal including oxygen saturation (SpO_2_) of 100% on ambient air. The intern was not able to appreciate jugular venous distention (JVD) or lower extremity edema but heard crackles at bilateral lung bases. He did not appreciate added sounds or murmurs on cardiac examination. Basic laboratory work-up showed mild normocytic anemia and elevated brain natriuretic peptide (BNP), as well high serum blood urea nitrogen (BUN), creatinine, and lactic acid. Chest X-ray (CXR) showed bilateral basal haziness “that might represent atelectasis versus consolidation,” as reported by radiologist.

The attending physician asked the residents about their assessment and management plan. Both the intern and the junior resident were not sure whether it was a case of pneumonia with hypovolemia and acute kidney injury (AKI) or heart failure (HF) and cardiorenal syndrome. Their argument for giving antibiotics and fluids was the possibility of the diagnosis of pneumonia with lactic acidosis and AKI in the setting of unknown baseline kidney function. They did not appreciate clear signs of overload (there was no leg edema or JVD, the patient’s SpO_2_ was normal, and the patient’s dyspnea and CXR findings could be from the pneumonia). Her elevated BNP, they said, could be from an unknown history of chronic rather than acute left ventricular (LV) dysfunction. On the other hand, their argument for HF and fluid overload was the patient's presentation of dyspnea, basal crackles, BNP elevation, and kidney dysfunction, which could be from poor cardiac output. The CXR findings in this situation could be interpreted as atelectasis, not pneumonia. The attending physician took the team to the bedside, confirmed their examination findings, and performed point-of-care ultrasound (POCUS). Plan and outcome of this case are delineated at the end of this review.

Why this topic is important

Assessment of patients’ volume status at the bedside is a very important clinical skill that physicians need in many clinical scenarios. Examples include patients with HF, chronic kidney and liver diseases, hyponatremia, sepsis, gastrointestinal (GI) bleeding, GI losses in the form of vomiting or diarrhea, and other situations [1-5].

Most of the studies that examined options for assessing and managing volume status were conducted in the ED and intensive care units (ICUs) [5-7], but clinicians in both the outpatient and inpatient settings need these skills and perform them frequently. For example, in a report by inpatient housestaff in one facility, 21% of total pages they received was reported to necessitate a volume status assessment [8].

Around 60% of our total body weight is water: two-thirds of this water is in the intracellular space and one-third is in the extracellular space, which can further be divided into interstitial space (which has 75% of extracellular space water) and intravascular space (which has the remaining 25%) [9]. Most clinicians refer to the extracellular component (and mostly to intravascular space) when making the diagnosis of hypovolemia (i.e., volume contraction) or hypervolemia (i.e., volume overload). Hypovolemia with hypotension and tissue under-perfusion is usually more alarming to physicians, but hypervolemia is also found to be associated with poor outcomes [10-16], making euvolemia a crucial clinical goal in patients suffering from any of these two conditions.

## Review

Assessing volume status at the bedside

In the absence of a gold standard test that is reliable and easily accessible to assist with decision-making, assessment of volume status can be challenging for physicians [17]. Many measures have been suggested to play the role of gold standard test given their increased sensitivity and specificity compared to history and physical examination (e.g., serum aldosterone and norepinephrine, bioelectrical impedance, radioisotope techniques to directly measure blood volume, central venous pressure [CVP], pulmonary artery catheterization to measure right atrial pressure and pulmonary capillary wedge pressure [PCWP], and transpulmonary thermodilution) [2,18-20]. Unfortunately, these are either invasive or widely unavailable at the bedside. In addition, there is a general perception that history and physical examination alone are insufficient to accurately assess volume status. A combination of history, physical examination, and bedside instruments is likely more useful, and some clinicians even advocate for the addition of scoring systems that combine elements from all of these instruments [21,22]. Therefore, using more than one evaluation method is important. Physicians need to have a broad knowledge of the strengths and limitations of these individual, non-invasive tools available at the bedside (Table 1).

**Table 1:** Non-invasive clinical instruments to assess volume status (summary of findings) POCUS, point-of-care ultrasound

Clinical Instruments	Comments
History and data gathering from the patient (primary source), or families, nurses, or electronic records (secondary source)	Presenting symptom(s), especially symptoms suggestive of volume loss (e.g., diarrhea, vomiting, bleeding, etc.)
	History of weight gain
	Past medical history (e.g., cardiac disease or history of heart failure)
	Cumulative fluid balance (care needs to be exercised to assure accuracy of data)
	Daily weights (can be helpful in patients with hypervolemia due to cardiac, renal, or liver disease)
	Urine output (oliguric kidney injury can be found in both hypovolemia and hypervolemia due to poor cardiac output)
Physical examination	Vital signs including postural blood pressure and pulse measurements (hypotension can be associated with both hypo- and hypervolemia due to cardiac dysfunction; interpret postural hypotension cautiously in the elderly; severe postural dizziness and postural increase of heart rate by 30 beats per minute can be useful indicators of hypovolemia; patients with renal disease are prone to both hypo- and hypervolemia)
	Examination of skin and mucous membranes (more reliable in children compared to the elderly; moist mucous membranes and axillae make dehydration less likely but do not rule it out; skin mottling is non-specific but can be associated with increased mortality in septic shock)
	Testing for capillary refill (might be useful only in severe hypovolemia)
	Neck examination for jugular venous distention (its presence can be useful to diagnose hypervolemia in heart failure patients, but its absence does not help make it less likely)
	Precordial auscultation for third heart sound (its presence can be useful to diagnose hypervolemia in heart failure patients, but its absence does not make it less likely; left lateral decubitus positioning might increase detection rate)
	Lung auscultation for basal crackles (non-specific; its presence only mildly increases the probability of heart failure in patients with dyspnea and its absence does not make it less likely)
	Lower extremity examination for edema (does not always indicate hypervolemia and it can absent in many patients with heart failure)
POCUS	Lung examination for congestion (presence of bilateral B-lines helps diagnose pulmonary edema and their numbers might correlate with degree of congestion; can be found in other interstitial processes)
	Inferior vena cava diameter and collapsibility (correlates with volume status more than physical examination; relatively easy to interpret by novice learners)
	Internal jugular vein distension (upper limit might be easier to see compared to physical examination; correlates with jugular venous pressure but might underestimate central venous pressure)
	Cardiac POCUS for left ventricular contractility using eyeball method (hypercontractility can be found in hypovolemia, and reduced contractility is found in patients with hypervolemia due to heart failure with reduced ejection fraction)
	Cardiac POCUS to estimate cardiac output (needs training and not easily feasible)
Common laboratory tests	Tests that can be associated with both hypo- and hypervolemia (serum sodium and osmolality changes; serum lactate elevation; serum blood urea nitrogen elevation)
	Serum uric acid (levels can be elevated in hemoconcentration associated with dehydration; levels below 4 mg/dL can be seen in euvolemic syndrome of inappropriate anti-diuresis)
	Urine studies (increased urine-specific gravity can be associated with dehydration but correlation is not always consistent)
	Natriuretic peptides (associated with hypervolemia; level below 100 pg/mL is highly reliable to exclude heart failure in patients with acute dyspnea)
Bioimpedance analysis	Not widely used or available

Patient history

Obtaining accurate history remains crucial in all clinical encounters, and assessing volume status is no exception. In one survey, the parameter most commonly used by inpatient housestaff for assessing volume status was a patient’s history [8]. Attention should be paid to cardiac history and daily fluid balance. A past medical history of HF increases the probability of diagnosing congestion and overload in patients presenting with dyspnea (positive likelihood ratio [LR+] of 5.8), but a history of paroxysmal nocturnal dyspnea only mildly increases that probability with LR+ of 2.6 [23]. HF patients are usually instructed to weigh themselves regularly, and a history of weight gain in these patients can be a useful indicator of hypervolemia. It is important to note that patients with diseases predisposing to tissue edema (including HF patients) can still present with relative effective hypovolemia if they have a history suggestive of volume loss (e.g., presented with acute diarrhea or GI bleeding).

For patients in the hospital setting, the recorded fluid balance or daily weights will aid in assessing volume status. Unfortunately, these records are not always available or accurate. In one survey, hospitalists and advanced practitioners reported that the day-to-day fluid balance recorded differed from the true value by as much as 1 liter [24]. In the same survey, 52% of respondents say that the most important findings used to assess adequacy of decongestion in patients admitted for HF is resolution of symptoms on exertion and they might take that as an indicator for consideration of discharge. In one study of 68 ICU patients, a history of fluid losses (e.g., aspiration of gastric content or drainage of chest or abdomen cavities) since ICU admission had the highest specificity for predicting hypovolemia [21]. Urine output is also important to note, although both hypovolemia and HF with hypervolemia can be associated with reduced urine volume. Interestingly, a patient’s report of thirst was not found to be related to dehydration severity [25].

Physical examination

Although the physical examination has long been a cornerstone of clinical decision-making, it is not without its limitations when it comes to volume status assessment since a number of physical signs have low sensitivity and specificity [26]. Certain populations such as patients with end-stage renal disease (ESRD) require highly objective measures to accurately assess “dry weight” due to their particular sensitivity to volume loads [27]. Despite these limitations, many examination findings reveal crucial insights into the underlying pathology affecting volume status and will be reviewed here.

Postural Pulse and Blood Pressure Changes

Change in blood pressure (BP) and pulse rate (PR) might be the first trigger for close evaluation of volume status in different clinical presentations. Orthostatic BP and PR measurements should be obtained when hypovolemia is suspected. Hypotension has a broad array of causes related to volume status, including GI or renal losses, sepsis and systemic infection, or severe cardiac dysfunction. The latter can present with seemingly contradictory signs of hypotension with peripheral edema secondary to reduced cardiac output and renal clearance; hence, a reduced BP does not always mandate immediate fluid replacement. Continuous saline infusion can lead to hypervolemia and can be a cause of hypertension, for example, in the perioperative period [28].

In the elderly, the presence of orthostatic hypotension should be interpreted cautiously due to both its high prevalence in this population and its low association with underlying pathology directly related to volume status. Prevalence in nursing homes can be as high as 50% and was not found to be associated with subsequent falls [29]. In the ED, wide variations in orthostatic measurements were found in patients who were presumed to be euvolemic [30]. However, a review by McGee et al. reported two valuable indicators of hypovolemia from severe hemorrhage: (1) severe postural dizziness precluding vitals checks on standing and (2) a postural increase in PR by 30 beats per minute [26]. This review also suggests that tachycardia when supine should be interpreted cautiously, for it has not been shown to be sensitive for hemorrhage even in large volumes.

Both hypovolemia and hypervolemia are common in patients with ESRD. Low BP in these patients is commonly attributed to hypovolemia, but that is not always the case. For example, rapid ultrafiltration can lower BP without the presence of hypovolemia. Also, patients with ESRD and LV dysfunction can have hypotension but still be hypervolemic [31].

Skin and Mucous Membranes

It is useful to note skin and mucous membrane examination findings in assessing volume status, but caution should be exercised. Elastin in skin depends on water to function properly, and patients with dehydration can have slow recoil when the skin is pinched between two fingers. Anecdotally, this sign is somewhat reliable in children but is less reliable in the elderly given the degeneration of elastin by the normal aging process [32,33]. The presence of moist mucous membranes, tongue, and axilla can make dehydration less likely. These signs also might correlate with dehydration severity regardless of age [25]. Dry axillary skin only mildly increases the probability of dehydration with a LR+ of 2.8 [26]. Skin mottling is a common finding in sick hospitalized patients but is non-specific and unlikely to help determine the cause of hypotension or shock. However, it was found to be associated with higher mortality when found in the pre-hospital setting in patients found to have septic shock [34,35]. Therefore, its presence can have prognostic value but does not assist in finding out the underlying cause of hypotension.

Capillary Refill

Capillary refill can be measured by pressing the distal phalange of the middle finger and then releasing it to calculate the time the blood returns to the finger bed. Classically, this happens within 2 seconds in men and up to 4 seconds in women. A delayed capillary refill might be useful only in severe hypovolemia but does not appear to be useful in mild-to-moderate degrees of blood loss [26,36]. When the test is done over the patella, the correlation was poor with the overall clinical suspicion of hypovolemia by ED physicians [25]. Just like the skin mottling sign, delayed capillary refill of more than 4 seconds was found to be associated with higher prehospital mortality in patients with septic shock [34].

Jugular Venous Distention

Using the appropriate bedside technique, the finding of raised JVD can be a useful examination finding in patients with hypervolemia and cardiovascular disease, although its absence does not reliably rule out congestion [37]. In a small study of 52 patients undergoing right heart catheterization, JVD - at rest or inducible - had the best combination of sensitivity (81%), specificity (80%), and predictive accuracy (81%) for elevation of PCWP (more than or equal to 18 mm Hg) among bedside cardiovascular examinations [38]. In the ESCAPE (Evaluation Study of Congestive Heart Failure and Pulmonary Artery Catheterization Effectiveness) trial, which examined the correlation between bedside clinical evaluation (history and examination) and more invasive hemodynamic measures using CPWP in advanced HF patients, elevated JVD and orthopnea were the only two bedside findings associated with an elevated PCWP [39]. Also, the finding of elevated JVD might be associated with worse outcome in HF patients [40]. In one review, JVD had a moderate LR+ of 5.1 in HF in patients with dyspnea [23]. However, the correlation of JVD with CVP has been questioned, and this physical sign can be challenging to elicit in some patients (e.g., obese patients and patients with short neck) [41].

Third Heart Sound

Third heart sound (S3) in one review had a relatively high LR+ of 11 for HF in patients with dyspnea [23], and just like elevated JVD it might be associated with worse outcomes [40]. However, it appears to have a low negative predictive value for high PCWP (more than 20 mm Hg) [42]. In another small study of 49 adults referred for evaluation of HF, the sensitivity and specificity of S3 for low ejection fraction (less than 50%) were 51% and 90%, respectively [43]. Therefore it appears that this sign is useful only when present. Also, it seems to require experience to be elicited [44]. Auscultating while the patient is in the left lateral decubitus position might assist in picking up this sign during cardiovascular examination if volume overload is suspected [45].

Lung Auscultation

Pulmonary basal crackles can be heard bilaterally in patients with pulmonary edema, but the usefulness of this finding to increase the pretest probability for this diagnosis is questionable. In one review, its presence only mildly increased the probability of HF in patients with dyspnea (LR+ 2.8) [23]. Another study with 43 patients with high jugular venous pressure measure by PCWP found that 18 (around 42%) of them did not have rales [46]. Therefore, not all patients with overload due to HF and high PCWP exhibit crackles [42]. Also, conditions other than pulmonary congestion can be associated with abnormal added sounds at the bases (e.g., atelectasis and pulmonary fibrosis).

Examination of Lower Extremities for Edema

In patients with low ejection fraction, lower extremity edema (and raised JVD) appears to perform better than other symptoms and signs of acute HF exacerbation [47]. Nevertheless, it appears that the presence of lower extremity edema does not always indicate hypervolemia. In one study on stable long-term hemodialysis patients, pretibial edema was found to correlate with obesity but not with relative blood volume, N-terminal prohormone B-type natriuretic peptide (NT-proBNP) levels or echocardiographic findings, suggesting that it might not be a good indicator of volume status in this population [48]. Edema was absent in close to half of patients with high jugular venous pressure estimated by PCWP in one study [46]. In non-ambulatory patients, edema will likely accumulate in the sacral area, which can be easily missed without careful examination. Fluid might also redistribute to this area, and improvement in leg edema on its own may not reflect overall improvement of congestion.

Point-of-care ultrasound

POCUS has evolved exponentially over the past decade. Ultrasound machines used to take up an entire room, now they can fit in physicians’ pockets and connect directly to tablets or smartphones, making them valuable bedside tools. Historically, used only in the ED and in critical care settings, POCUS is now gaining popularity in internal medicine with the advent of portable devices [49,50]. It is also a skill that is safe, quick, and approachable for most students, and is associated with more patient satisfaction in the ED [51-53].

POCUS is now suggested or endorsed by many internal medicine societies (e.g., the Society of Hospital Medicine, the Association of European Society of Cardiology, and, recently, the American College of Physicians) to assist physicians with diagnosing and treating many common pathologies [54-56]. Several studies showed that medical residents are interested in POCUS and therefore many medical schools have started POCUS curricula [57,58]. Although ultrasound is operator-dependent and clinical utility is expected to improve with more practice, POCUS is accessible to students at all training levels and can be performed by non-physicians and physicians-in-training with sensitivity and specificity exceeding that of CXR for pathologies such as pulmonary edema and pleural effusion [51,59].

As a bedside tool, POCUS seems to be promising for volume status assessment. Images and clinical information can be obtained relatively quickly from different organs including the lungs, heart, and large vessels to inform medical decisions regarding fluid management. We will discuss some of these useful bedside applications.

Lung POCUS

Lung ultrasound (LUS) technique typically includes examining eight lung regions (for on each side; known as the BLUE protocol) [60]. Contrary to other radiological modalities, artifacts are paradoxically useful in LUS. LUS artifacts are used to diagnose a wide array of diseases in the lung, including pulmonary edema and pleural effusions in patients with hypervolemia and pulmonary congestion. One of these artifacts, called B-lines, is a reverberation artifact that is suggestive of pulmonary edema and can be very useful when hypervolemia is suspected [61,62]. B-line artifacts, as described by Soldati and Demi, can be defined as “laser‐like vertical hyperechoic reverberation artifacts that arise from the pleural line, extend to the bottom of the screen ‘without fading irrespective of the programmed depth of penetration’, and move synchronously with the respiratory cycle” [63]. The presence of bilateral, diffuse B-lines during LUS examination is highly suggestive of cardiogenic pulmonary edema, although this can also be seen in some other diseases such as acute respiratory distress syndrome and bilateral interstitial pneumonia, e.g., coronavirus disease 2019 (COVID-19) [64].

In patients with hypervolemia due to acute decompensated HF, LUS in one review was more sensitive and specific than CXR in looking for features of pulmonary congestion, and in another meta-analysis it outperformed CXR in the detection of pleural effusions [52,65]. It was also found to be very useful to diagnose HF in patients presenting to the ED with dyspnea, even when compared to NT-proBNP [66]. In one meta-analysis, the sensitivity and specificity of LUS in acute cardiogenic pulmonary edema were around 92% and 94%, respectively [67]. The European guidelines stated that “bedside thoracic ultrasound for signs of interstitial edema and pleural effusion may be useful in detecting acute HF if the expertise is available” [68].

Not only the presence of lung B-lines is helpful, but the number of these artifacts can also be associated with the degree of overload, and a reduction in number is seen after removal of excess fluid [69,70]. Combined with natriuretic peptides, LUS can assist in diagnosing pulmonary edema in cases where these tests are suspected to be falsely low (e.g., obesity) [69]. It has also shown usefulness in the outpatient setting where its use was associated with reduced visits to urgent care clinics due to worsening HF and fluid overload [71].

POCUS of the Inferior Vena Cava

Inferior vena cava (IVC) diameter and collapsibility during inspiration can be easily obtained by using cardiac or abdominal probes at the subcostal area close to the midline. This examination was found to correlate fairly with volume status and performs more accurately than physical examination. In one small observational study, blood loss of as low as 450 mL decreased the IVC diameter by a mean of around 0.5 cm in 31 volunteers after blood donation [72]. Another study showed that in a small number of hemodialysis patients, IVC examination was useful in detecting dry weight and can also help determine the ultrafiltration volume [73,74]. European guidelines also support the use of IVC POCUS in assessing volume status in HF patients and gave it class IIb recommendations [68]. The IVC examination can be part of limited echocardiographic assessments in the ICU for patients with shock, and this assessment was found to be associated with improved outcomes when used to guide fluid therapy following early resuscitation in early shock state [75]. It can also help assess volume status in decompensated liver cirrhosis and in HF patients in the outpatient setting where labs and imaging are not readily available [76,77]. This examination appears to be relatively easy to acquire and interpret at the bedside by novice learners. In one survey, medical residents reported that they feel more confident performing IVC POCUS examination than any other POCUS skills, and during an image interpretation test it ranked first [57].

POCUS of the Internal Jugular Vein

Similar to IVC POCUS principles, a relatively new POCUS skill can also be useful for volume status assessment: examination of the internal jugular vein (IJV) for raised JVD [78]. It is done in a similar fashion to the traditional neck examination for JVD, but this time the height of the JVD can be easily seen with ultrasound to see if JVD is elevated or not. Using a vascular or high-frequency probe, the examiner can start at the base of the right lateral neck to identify the IJV, first in cross-section view then longitudinally, and then slowly slide up the IJV course to determine the height of the JVD by finding the “meniscus” where the opposing walls of the IJV meet or near meet, especially during inspiration. This can be particularly useful in patients whose neck examinations are perceived to be challenging due to obesity or short neck. More detailed measurements of JVD using ultrasound were also found to correlate with higher serum NT-proBNP levels and also worse outcome in HF patients in outpatient settings [79,80]. In one prospective study in the ICU setting, JVD by ultrasound accurately reflected jugular venous pressure but underestimated CVP [81].

POCUS of the Heart

Cardiac POCUS can be learned relatively quickly by residents and non-cardiologists and can improve their clinical assessment of LV function among other clinical cardiac bedside skills [82,83]. The eyeballing method is reliable in estimating the LV function as hypercontractile, normal, reduced, or severely reduced [84]. Hypercontractility of the LV can be found in patients with hypovolemia, and reduced contractility is found in patients with hypervolemia due to LV dysfunction. This can be very useful in patients with hypotension to differentiate between hypovolemic and cardiogenic shocks [84,85].

In patients suspected to have hypovolemia, using cardiac POCUS to measure stroke volume (using the left parasternal and the five-chamber views) before and after a leg raising test might be helpful in determining which patients can benefit from fluid challenge [86-88]. However, compared to other POCUS skills, this one requires more advanced training and is not routinely performed by most internal medicine physicians who use POCUS.

Laboratory tests

There is no “gold standard” laboratory marker for determining volume state. A set of readily available lab tests, when combined with adequate history, physical examination, and POCUS, can shed light on both the volume state and its etiology. Therefore, an understanding of laboratory tools is paramount for a timely assessment of hydration status. Here we will discuss basic labs that are widely used and readily available to internists.

Serum Sodium and Plasma Osmolality

Fluid balance in the human body is generally determined by the sodium (Na+) ion, and when abnormalities in volume status develop, they typically manifest as irregularities in the serum Na+ concentration. Hypo- and hypernatremia are generally defined by [Na+] levels <135 or >145 mEq/L, respectively, with the latter typically seen as an indicator of dehydration due to water loss being the predominant cause in the vast majority of cases. Hyponatremia, on the other hand, has a much broader etiology and can be associated with any volume state [89,90]. Similarly, higher plasma osmolality is generally seen in fluid depleted states whereas lower plasma osmolality can be associated with any volume state [91]. Therefore, the utility of plasma osmolality or Na+ concentration alone is limited when elucidating hydration status.

Serum Lactate

Lactate is a byproduct of anaerobic metabolism. Although the causes of lactic acidosis are many, the vast majority are type A (disorders associated with tissue hypoxia) arising from hemodynamic compromise, namely, cardiogenic/hypovolemic shock, severe HF, or sepsis. In contrast, Type B lactic acidosis is not caused by tissue hypoxia and has a broad array of causes less related to volume status than type A, ranging from carbon monoxide poisoning to even thiamine deficiency [92]. Most laboratories employ a normal range for lactate of 0.5 to 2.2 mmol/liter, and additional findings supportive of hypovolemic compromise as a cause of lactic acidosis include a blood pH of ≤ 7.35 and a serum [HCO3-] of ≤ 20 mmol/liter [93]. However, the absence of the latter two findings does not necessarily rule out the presence of lactic acidosis nor a hypovolemic state. In fact, concurrent metabolic alkalosis as a result of volume loss can be seen, resulting in an elevated lactate with an alkalotic pH and hyperbicarbonatemia. Additional clue from the patient’s history suggestive of hypovolemia in the setting of lactic acidosis with concurrent metabolic alkalosis is recent diuretic use or GI losses of Na+ and chloride, which hints toward a contraction alkalosis and resulting hemodynamic compromise [94]. Indeed, this constellation of laboratory findings - lactic acidosis with contraction alkalosis - can paint a characteristic picture of a hypovolemic state with marked tissue hypoxia.

Serum Blood Urea Nitrogen and Serum Creatinine

Elevated BUN and creatinine can be found in both hypovolemia from dehydration and hypervolemia from HF causing impaired renal perfusion. In fact, in patients with HF, BUN elevation might be associated with worse prognosis, similar to NP-proBNP [95]. Patients with hypovolemia due to upper GI bleeding also might have elevations of BUN due to digestion of blood proteins, and this might indicate severe bleeding [96]. Serum BUN may not increase much in patients with liver disease or protein malnutrition.

Serum Uric Acid

Primarily synthesized in the liver, intestines, and endothelium as the end product of amino acid degradation, uric acid serves as the primary antioxidant agent in plasma that scavenges reactive oxygen species and peroxynitrites [97]. Indeed, 90% of the freely filtered uric acid from renal glomeruli, which removes 70% of circulating levels, is reabsorbed, which supports its important physiological role. It is normally soluble in human blood at concentrations up to 7 mg/dL, beyond which its pathological potential in the form of gout can become apparent when it begins depositing in connective tissues as urate crystals. Serum uric acid > 5 mg/dL can serve as an additional potential marker of hypovolemia in patients with hyponatremia [98]. Although hyperuricemia is technically defined as serum uric acid levels >12 mg/dL, an elevation above 7 mg/dL (although some experts suggest values as low as 6 mg/dL) with other supporting examination findings and laboratory markers can be considered abnormal given the risks of urate crystal deposition, and hemoconcentration from hypovolemia should be considered. In contrast, levels below 4 mg/dL can be seen in euvolemic syndrome of inappropriate anti-diuretic hormone (SIADH) secretion due to ADH-induced water retention and subsequent hemodilution, as well as increased excretion of urate in urine along with natriuresis as a compensatory mechanism of unknown etiology [99-101].

Urine Studies

Although not a part of most routine urinalyses ordered on first encounter, urine osmolality in addition to specific gravity are simple tests that can be quickly determined from most urine samples. A generally accepted threshold of dehydration for urine osmolality is ≥700 mOsm/kgH_2_O and urine-specific gravity > 1.020, although some studies recommend >1.025 for individuals with higher muscle mass, such as athletes [102,103]. However, caution should be exercised when interpreting urine studies alone without consideration of other laboratory or examination findings, for many studies have failed to show a consistent correlation between urinary markers of volume status and plasma osmolality or sodium concentrations, which are generally regarded as more reliable indicators [104,105]. Indeed, urine production and collection is subject to many confounding variables, such as time since the last void and individual biological variation in urine excretion rates; thus, clinical judgment is warranted when interpreting urine studies for volume assessment purposes [106].

Natriuretic Peptide

Unlike the aforementioned biomarkers, which could suggest volume-depleted or overloaded states, natriuretic peptides are best suited for ruling out hypervolemia. Both atrial natriuretic peptide (ANP) and BNP are synthesized in the heart - ANP in the atria and BNP in the ventricles - and are generally indicative of excessive volume pressure in the setting of LV dysfunction [107]. Their usefulness, however, is mostly found in ruling out hypervolemia. Serum level below 100 pg/mL for BNP has been demonstrated to be highly reliable for excluding HF, but above this value the usefulness of BNP diminishes due to its lack of specificity [23]. Furthermore, BNP is known to remain consistently elevated for several days after resolution of hypervolemia in patients with HF. In contrast, ANP has a much shorter half-life, and its levels fluctuate quickly with volume status in dialysis patients, remaining persistently elevated in overload and dropping quickly when hypovolemic as assessed by bioimpedance [108-110]. Nevertheless, from a practical standpoint, BNP remains more widely used due to its broad assay availability and recognition as a marker of HF than ANP.

In addition to BNP, its posttranslational byproduct NT-proBNP is also available as a marker for both HF and volume overload [111]. Once released by ventricular myocytes in response to pressure or volume overload, the 134 amino acid pre-pro-BNP peptide is cleaved into the biologically active 32 amino acid BNP and the 76 amino acid N-terminal NT-pro-BNP. Only BNP has a known natriuretic effect by counteracting the renin-angiotensin-aldosterone system and by vasodilation, but both peptides are detectable in plasma soon after release. However, controversy exists over its diagnostic concordance with BNP despite its widespread acceptance as being clinically equivalent. A recent study by Farnsworth et al, testing BNP vs NT-proBNP on 3,029 patients showed low diagnostic concordance between the two markers at their current cut-offs for HF (100 vs 300 pg/mL for BNP and NT-proBNP, respectively), and concordance was further decreased by renal disease [112]. Furthermore, the International Federal of Clinical Chemistry (IFCC) Committee on Clinical Applications of Cardiac Biomarkers advises against using different natriuretic peptide assays for clinical testing [113]. Therefore, although the natriuretic peptides can be easily screened for HF and indirect volume status evaluation, care should be exercised by the clinician in their selection and interpretation.

Bioimpedance analysis

A less commonly known yet highly effective non-invasive tool for assessing volume status (although not widely clinically available in practice) is bioimpedance analysis (BIA). BIA exploits the difference in resistive indices between extra- and intracellular water to calculate approximate volume status when a current is applied [114]. In general, a patient is considered “hypervolemic” when extracellular fluid (ECF) is found to be >105% of a calculated ideal and hypovolemic when <95% of ideal. In hemodialysis where volume control is crucial, BIA has proven to be a superior and objective assessment of fluid status versus BP and weight monitoring through many studies [115,116]. However, this modality is not without its limitations. Aside from requiring operator training, BIA results can be skewed by multiple factors including pregnancy, young age (children), presence of a pacemaker, peritoneal dialysis, and morbid obesity, and also cannot distinguish plasma volume from ECF, making BIA incapable of calculating intravascular volume [117,118]. Furthermore, some studies found up to 21% of patients originally believed to have excess ECF by BIA to actually be hypovolemic by clinical assessment. Although BIA is invaluable for volume assessments in dialysis, its applicability to other clinical settings remains to be seen.

Back to the case

The POCUS examination images obtained by the treating team are shown in Videos 1-6. Reviewing all the data obtained from the history, physical examination, POCUS, and laboratory tests, the team agreed that new-onset HF is the most likely diagnosis: an elderly female with fatigue, cough, dyspnea, and basal crackles; POCUS with reduced LV contractility, dilated IVC, bilateral pleural effusions, and bilateral pulmonary B-lines; elevated BNP; and impaired kidney function. This is the most likely diagnosis despite the absence of history of cardiac disease or atherosclerotic risk factors (apart from age), leg edema, JVD, S3, or hypoxia. The patient was admitted and started on intravenous diuretic therapy and HF therapy. Her symptoms improved, as well as her serum BUN, creatinine, and lactic acid. Echocardiography was obtained confirming the diagnosis of HF with reduced ejection fraction, and she was discharged with outpatient follow-up with cardiology for the evaluation of ischemic heart disease as the possible underlying cause of new HF diagnosis.

**Video 1:** Point-of-care cardiac ultrasound (long axis left parasternal view) showing reduced left ventricular contractility

**Video 2:** Point-of-care ultrasound showing a large inferior vena cava that does not collapse with inspiration

**Video 3:** Point-of-care ultrasound of the right upper lung anteriorly showing multiple B-lines

**Video 4:** Point-of-care ultrasound of the left upper lung anteriorly showing multiple B-lines

**Video 5:** Point-of-care ultrasound of the right lung base posteriorly showing moderate pleural effusion

**Video 6:** Point-of-care ultrasound of the left lung base posteriorly showing small pleural effusion

## Conclusions

Assessment of patient’s volume status is a common and important clinical conundrum that is frequently encountered in both inpatient and outpatient settings. Unfortunately, no gold standard test is available for an expedient answer to the question of whether a patient is hypovolemic, euvolemic, or hypervolemic. However, modern clinicians do have a wide array of proven tools and emerging technologies for fast assessments. The highest yield will come from examining all the data from history, physical examination, and laboratory tests, rather than relying on individual information or signs. POCUS is a promising sensitive and specific tool that can provide a lot of clinical information at the bedside to supplement the physical examination.
